# Proteomic Analysis of the Mediator Complex Interactome in *Saccharomyces cerevisiae*

**DOI:** 10.1038/srep43584

**Published:** 2017-02-27

**Authors:** Henriette Uthe, Jens T. Vanselow, Andreas Schlosser

**Affiliations:** 1 Rudolf Virchow Center for Experimental Biomedicine, University of Wuerzburg, Josef-Schneider-Str. 2, 97080, Wuerzburg, Germany

## Abstract

Here we present the most comprehensive analysis of the yeast Mediator complex interactome to date. Particularly gentle cell lysis and co-immunopurification conditions allowed us to preserve even transient protein-protein interactions and to comprehensively probe the molecular environment of the Mediator complex in the cell. Metabolic ^15^N-labeling thereby enabled stringent discrimination between *bona fide* interaction partners and nonspecifically captured proteins. Our data indicates a functional role for Mediator beyond transcription initiation. We identified a large number of Mediator-interacting proteins and protein complexes, such as RNA polymerase II, general transcription factors, a large number of transcriptional activators, the SAGA complex, chromatin remodeling complexes, histone chaperones, highly acetylated histones, as well as proteins playing a role in co-transcriptional processes, such as splicing, mRNA decapping and mRNA decay. Moreover, our data provides clear evidence, that the Mediator complex interacts not only with RNA polymerase II, but also with RNA polymerases I and III, and indicates a functional role of the Mediator complex in rRNA processing and ribosome biogenesis.

The Mediator complex is an essential coactivator of eukaryotic transcription. Its major function is to communicate regulatory signals from gene-specific transcription factors upstream of the transcription start site to RNA Polymerase II (Pol II) and to promote activator-dependent assembly and stabilization of the preinitiation complex (PIC)^1^,^2^,^3^. The yeast Mediator complex is composed of 25 subunits and forms four distinct modules: the head, the middle, and the tail module, in addition to the four-subunit CDK8 kinase module (CKM), which can reversibly associate with the 21-subunit Mediator complex. Whereas yeast Mediator without the CKM is generally associated with transcriptional activation, presence of the CKM is typically associated with transcriptional repression^4^.

There is growing evidence that the Mediator complex is also involved in post-initiation stages of Pol II transcription^5^,^6^. In budding yeast, a functional role of Mediator was uncovered for transcription elongation^7^,^8^,^9^, transcription termination^10^,^11^, as well as for mRNA export^12^. Furthermore, genome-wide location analyses revealed Mediator interactions with coding regions^13^,^14^, although these findings are controversial^15^,^16^. In addition, the yeast Mediator tail-module has been shown to be required for telomere heterochromatin maintenance and is involved in telomere length regulation^17^.

Mass spectrometry contributed in various ways to our current knowledge about the Mediator complex. E.g., mass spectrometry was applied to determine the components of yeast Mediator complexes^18^, or in combination with chemical cross-linking to support cryo-electron microscopy studies of the yeast Mediator complex^19^,^20^. A number of interaction partners of the Mediator complex were identified by large-scale mass spectrometry-based interactome analyses in yeast^21^,^22^ as well as in human cell lines^23^,^24^. However, the overall number of Mediator interaction partners identified from these studies is limited. We think this is mainly due to the cell lysis and CoIP conditions applied in these studies, which are suboptimal in preserving transient interactions. E.g., in both yeast studies the classical tandem affinity purification (TAP) strategy has been applied, that due to its multi-step procedure tends to discriminate against transient interactors.

In order to obtain a more complete picture of the various cellular functions of the Mediator complex and in order to identify potential regulators of the Mediator complex we performed a comprehensive analysis of the yeast Mediator complex interactome.

## Results

We applied co-immunopurification (CoIP) with HA-tagged endogenous Mediator subunits in combination with quantitative mass spectrometry for identifying *in vivo* interaction partners of the yeast Mediator complex. Yeast cells were lysed by cryogenic grinding with liquid nitrogen in a planetary ball mill according to a protocol introduced by the Rout lab^25^. This method is very efficient in disrupting yeast cells and at the same time gentle enough to preserve even labile protein complexes. Cryogrinding turned out to be a critical step for the successful identification of transient and labile interaction partners. The Mediator complex does not directly bind to DNA, but is known to interact with various DNA-binding proteins, such as activators and RNA polymerases. In order to ensure the detectability of such interacting DNA-binding proteins, we applied a non-specific endonuclease during CoIP. Initially, we tested a number of different HA-tagged subunits and found that experiments with C-terminally HA-tagged Med17 resulted in the highest number of identified specific interaction partners. For distinguishing such *bona fide* interaction partners from nonspecifically captured proteins, we performed parallel negative control CoIPs from metabolically ^15^N-labled wild type cells under the same conditions, pooled the eluates from both CoIPs and analyzed the mixture by GeLC-MS/MS^26^. The whole experiment was conducted in three independent biological replicates.

**Figure 1:**
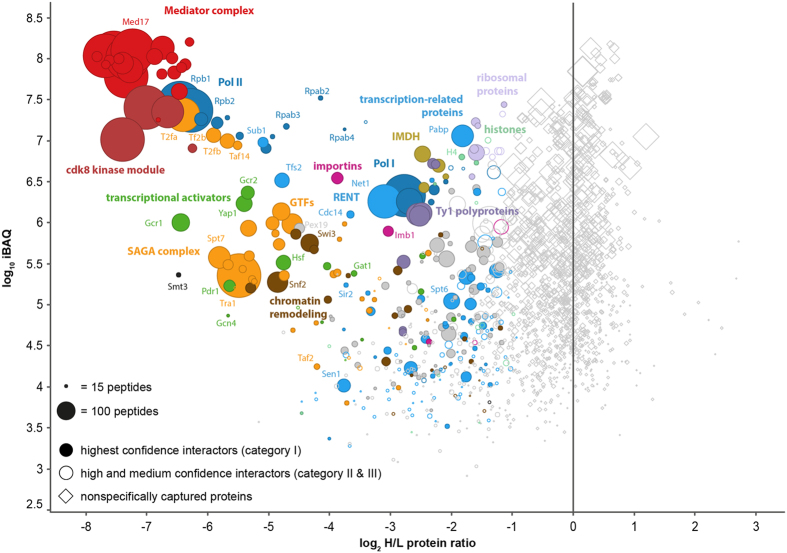
Yeast Mediator complex interactome derived from CoIPs with Med17. CoIPs with Med17-HA were performed from cells grown in light medium (^14^N, light), control CoIPs with wild type cells were performed in parallel (mixing after purification, MAP) from metabolically labeled cells (^15^N, heavy). The plotted log_2_-transformed heavy-to-light (H/L) protein ratios and log_10_ iBAQ values are median values calculated from three biological replicates. Nonspecifically captured proteins have a log_2_ H/L protein ratio around 0, whereas specific interaction partners show up with negative log_2_ H/L protein ratios. The size of a data points correlates with the number of peptides identified for the corresponding protein. Proteins from the same complex, and proteins with related functions are depicted in the same color. The complete data set is given in Supplemental Table S1.

The particularly mild cell lysis and CoIP conditions allowed us to identify an extraordinary large number of specific interactors of the Mediator complex. In total we identified 467 proteins as specific interaction partners of the Mediator complex (Fig. 1, Table 1 and Table S1), from which we classified 228 as highest confidence interactors (category I), 167 as high confidence interactors (category II), and 72 as medium confident interactors (category III) (details of categorization see Experimental Procedures and Figure S1). Many of these proteins have not been identified as interactors of the Mediator complex before. Only 68 of the 466 interactors are listed as highly confident interactors in the HitPredict database (Figure S2 and Table S1), a meta database for protein-protein interactions^27^.

In addition to the H/L protein ratio, which allows to distinguish between *bona fide* interactors from nonspecifically captured proteins, we used the iBAQ (intensity-based absolute quantification) value^28^ as a second quantitative parameter to characterize the identified proteins. The iBAQ value can be used as a measure for the absolute protein amount and allows to estimate interaction stoichiometries^23^,^29^. Subunits of a protein complex and other stoichiometric interaction partners tend to have similar H/L protein ratios as well as similar iBAQ values and thus typically cluster together in a scatter plot of both values. This becomes apparent for example for the 21 subunits of the Mediator complex shown in Fig. 1. The four subunits of the CKM (Srb8, Ssn2, Ssn3, and Ssn8) show significantly lower iBAQ values and clearly separate from the 21 Mediator subunits, indicating substoichiometric binding of the kinase module to the Mediator complex. Based on the iBAQ values of Mediator (median iBAQ for the 21 core subunits: 100 × 10^6^) and CKM (median iBAQ for the 4 CKM subunits: 15 × 10^6^) we estimate that about 15% of the Mediator are in complex with the kinase module. Similar high iBAQ values as for the kinase module are observed for all 12 subunits of Pol II (median iBAQ for the 12 Pol II subunits 15 × 10^6^), suggesting that another 15% of the Mediator are in complex with Pol II. Since binding of the kinase module and of Pol II are mutually exclusive^30^, we estimate that about 70% of the Mediator complex exists as free Mediator, which is in good agreement with results reported by Takagi *et al*.^31^ and Rani *et al*.^32^.

A recent study indicates that a Mediator subcomplex consisting of Med2, Med3 and Med15 exists^33^. The existence of this or any other subcomplex, apart from Mediator without CKM does not get evident from our stoichiometric data. However, our data does also not exclude the existence of such subcomplexes. If present in relatively low amount, the presence of subcomplexes would probably not be detectable from our global iBAQ-based analysis.

### Proteins of the PIC and Activators are the Most Prominent Interactors of the Mediator Complex

Pol II is one of the most prominent interactors identified in our interactome analysis. All 12 subunits of Pol II are detected with high iBAQ values, indicating that Pol II is, as expected, one of the most abundant interaction partners of Mediator. Additional interactors identified with a similar high interaction stoichiometry as Pol II are the three subunits of TFIIF (T2fa, T2fb and Taf14), TFIIB (Tf2b), and Sub1 (Fig. 1). All these proteins are functional components of the PIC and are known interactors of Mediator. For Rpb3, a direct interaction with Med17 has been demonstrated by means of *in vivo* photo crosslinking^34^, and in a recent study from the Cramer lab it has been shown by chemical crosslinking that Rpb1,2,3,4, Rpb11, and T2fa are direct interactors of the Mediator, and that T2fb and Tf2b interact with Mediator via one additional protein^35^.

Sub1 is a ssDNA-binding protein that binds to Pol II and Pol III promoters^36^,^37^ and associates with Pol II at different stages of transcription^38^. Because Sub1 shows a similar interaction stoichiometry as direct interactors of Mediator, we conclude that Sub1 is also either a direct or very close interactor of the Mediator. All other components of the PIC, except for TFIIA are identified as specific interactors. In contrast to TFIIB und TFIIF however, TFIID (including TATA-box binding protein), TFIIE, TFIIH and the Spt–Ada–Gcn5 histone acetyltransferase (SAGA) complex (Table 1) are detected with lower interaction stoichiometries (Fig. 1).

In addition to essentially all components of the PIC, our interactome analysis identifies 18 different transcriptional activators (see Table 1), such as Gcr1, Gcr2, Gcn4, Yap1, and Hsf, as specific interaction partners of the Mediator complex. Only three of these proteins (Yap1, Pdr1 and Gcn4) have been previously reported as high confident interaction partners of Mediator in the HitPredict database so far. Gcr1 and Gcr2, which regulate expression of glycolytic and ribosomal genes show together with Yap1 the highest interaction stoichiometries (Fig. 1). In addition, we identify MCB-binding factor (MBF), composed of Swi6 and Mbp1, as specific interactor. MBF is a cell-cycle dependent transcription factor that regulates transcriptional activation of genes in late G_1_ phase^39^.

### The Mediator Interactome Indicates Cellular Functions of Mediator Beyond Transcription Initiation

The identification of transcriptional activators and essentially all components of the PIC as prominent interaction partners of the Mediator complex reflect Mediator’s well-characterized function in promoting activator-dependent assembly and stabilization of the PIC. In addition to activators and components of the PIC, we identified a number of interactors that are involved in Pol II elongation and in co-transcriptional processes. One example is TFIIS (Tfs2) (Fig. 1), an important transcription elongation factor^40^. However, since TFIIS also seems to play a role during transcription initiation^41^, we cannot conclude whether Mediator interacts with TFIIS during initiation, during elongation, or during both stages. In addition to TFIIS, we identified the histone chaperones Spt6 and the two components of the FACT (FAcilitates Chromatin Transcription) complex, Spt16 and Pob3. Both histone chaperones are important to facilitate Pol II transcription of nucleosome-covered DNA^42^. Spt6 has previously also been identified in a large-scale interactome study as an interactor of the Mediator^21^. The polyadenylate-binding protein (PABP) (Fig. 1), proteins involved in mRNA splicing (Brr2, Cbp2), mRNA decapping (Dcp2, Edc3), and mRNA decay (Xrn1) are additionally identified interactors. Taken together, our interactome analysis further supports the recent findings that Mediator plays a functional role in transcription elongation^7^,^8^,^9^ and in mRNA processing^6^.

For a few prominent interactors, namely Pex19, inosine-5′-monophosphate dehydrogenase (IMDH), importins and Ty1 polyproteins (Fig. 1, Table 1 and Table S1) a functional role in transcription or co-transcriptional processes is not directly evident. These proteins might have a yet uncovered function in transcription. Pex19 for example is a chaperone and import receptor for newly-synthesized peroxisomal membrane proteins and is first of all not expected to be involved in transcription. However, Pex19 has recently been identified as a strong transcriptional activator^43^, indicating that this protein might have a moonlight function in transcription. Similar, IMDH, of which four different isoforms (Imdh1-4) have been identified as specific interaction partners of Mediator has been recently described as a protein that binds nucleic acids *in vitro* and *in vivo*^44^, making a functional role of this protein in transcription conceivable.

### Mediator Interacts with Pol I and Pol III and with Proteins Involved in Co-Transcriptional Processing of rRNA and tRNA

To our surprise, we did not only identify all 12 subunits of Pol II, but also all 14 subunits of Pol I as specific interaction partners of the Mediator complex (Fig. 1, Table 1, Table S1). The interaction stoichiometry of Pol I is about one order of magnitude lower compared to that of Pol II. An interaction between Mediator and Pol I is further supported by a study showing that SAD1, an RNA polymerase I subunit of rice, interacts with Mediator^45^.

In contrast to Pol II, not many of the Pol I-specific initiation factors are identified as interactors of the Mediator. Initiation by Pol I requires Rrn3, upstream activating factor (UAF), and core factor (CF). From these, only one subunit of UAF (Rrn5) is identified as a specific interactor. However, the high-mobility group protein Hmo1 that binds to actively transcribed rDNA^46^ as well as to the promoter of most ribosomal protein genes^47^ is identified as a prominent interaction partner. In addition, we identified all three constituents of the RENT (regulator of nucleolar silencing and telophase exit) complex (Net1, Cdc14 and Sir2) as specific interactors. Net1 directly binds Pol I and stimulates rRNA synthesis *in vitro* and *in vivo*^48^. According to their iBAQ values, the RENT complex and Hmo1 appear as stoichiometric interactors of Pol I. Moreover, we were able to identify a number of proteins involved in rRNA processing and ribosome biogenesis (Table 1).

In addition to all Pol I subunits we also identified the two large subunits of Pol III (Rpc1 and Rpc2) together with components of the Pol III initiation factors TFIIIB and TFIIIC as interactors of Mediator (Table 1, Table S1). This finding is supported by a recent study describing that loss of Med20 affects transcription of tRNA and other non-coding genes in fission yeast^49^, indicating that Mediator might indeed play a functional role in regulating Pol III transcription.

In summary, these results clearly indicate that Pol I and III are functional interactors of the Mediator complex, and that Mediator in analogy to mRNA transcription seems to play a role in co-transcriptional processes of rRNA and tRNA transcription.

### Mediator Interacts with Chromatin Remodeling Complexes and with Highly Acetylated Histones

Chromatin remodeling is an integral part of transcriptional activation, and a functional relation between Mediator and chromatin remodeling has been demonstrated^50^. Furthermore, the SWI/SNF chromatin remodeling complex has been co-immunopurified with Mediator^51^. In addition to the SWI/SNF complex, our interactome analysis revealed also interactions with chromatin remodeling complexes of the ISWI, as well as the INO80 family (Table 1). However, the highest interaction stoichiometries were obtained for subunits of the SWI/SNF remodeling complex. Although described as a component of the SAGA complex^52^, Chd1, the only member of the CHD family of chromatin remodeling complexes in yeast, was not detected in our experiments. A recent mass spectrometric analysis of the SAGA complex also failed to detect Chd1 for unknown reasons^53^. In addition to chromatin remodeling complexes and to histone chaperones, we identified histones H2A, H2B, H3, H4 and H2A.Z as specific interaction partners of the Mediator complex. This is in line with the recent finding that Mediator directly interacts with the N-terminal tail of histone H4^54^. Furthermore, we have observed that all interacting histones are highly acetylated at their N-termini (Figure S3). In contrast to *in vitro* peptide binding assays, in which H4K16 acetylation impairs Mediator-H4 interaction^55^, we co-immunopurified H4 predominantly acetylated at K16 (Figures S3 and S4). In addition to the large number of acetylation sites, we detected mono-, di-, and trimethylation on H3K36 and on H3K79 (see Figure S3). Methylation on each of these sites correlates with active transcription^56^,^57^. Other types of modifications have not been detected on the co-purified histones.

### The Yeast Mediator Interactome is Highly Dynamic

**Figure 2:**
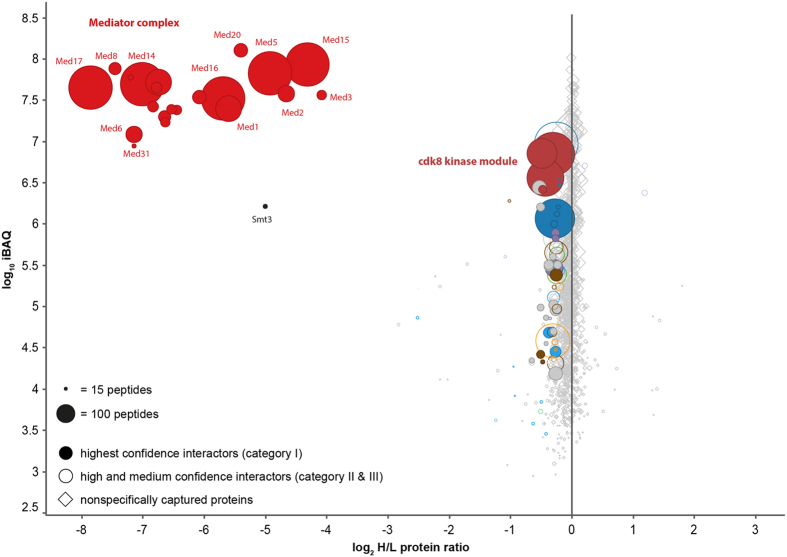
Results of a purify-after-mixing experiment (PAM) with Med17. Metabolically ^15^N labeled cells expressing Med17-HA were mixed with wild type cells grown in light medium, and CoIPs were performed after cryogrinding under the same conditions as for the MAP experiment shown in Fig. 1. The plotted log_2_-transformed heavy-to-light (H/L) protein ratios and log_10_ iBAQ values are median values calculated from three biological replicates. Stable interactors are identified predominantly in their ^15^N-labeled form, whereas transient interactors can exchange during CoIP and show log_2_ H/L protein ratios close to 0. Essentially all interaction partners of Mediator have log_2_ H/L protein ratios close to 0 and are therefore classified as transient interactors. Only Smt3 (SUMO) did not exchange in this experiment, indicating covalent binding to one or several subunits of Mediator.

In order to differentiate systematically between stable and transient interactors, we performed a so-called purification-after-mixing (PAM) experiment^58^. To this end we mixed Med17-HA cells grown in light medium with wild type cells grown in heavy medium (^15^N) and performed cryogrinding and CoIP under the same conditions as for the mixing-after-purification (MAP) experiment shown in Fig. 1. Since a PAM experiment is performed with the same amounts of light and heavy proteins, transient interactors, which exchange during CoIP will have H/L protein ratios approaching 1, in contrast to stable interactors that do not exchange. Figure 2 shows that, except of the 21-subunit Mediator complex itself (i.e. the head, middle and tail module), essentially all interactors of Mediator, including the CKM have a H/L ratio of close to 1 and are almost completely exchanged during the two hours of CoIP (see also Table S2). This indicates that the interactome of the Mediator complex is highly dynamic. Even the subunits of the head, middle and tail module of Mediator have started to exchange and show less negative H/L ratios compared to the MAP experiment. Especially subunits of the tail module (Med2, Med3, Med5, Med15, Med16) show up with H/L ratios significantly shifted towards 1, indicating that the interaction of the tail module with the rest of Mediator is more dynamic than the interaction between the other modules. These results are in line with the recent finding that a subcomplex consisting of Med2, Med3 and Med15 exists, that can be independently recruited to regulatory regions of genes^33^.

The only protein apart from the 21 subunits of Mediator’s head, middle and tail module that is identified with a H/L protein ratio significantly different from 1 in the PAM experiment is Smt3 (SUMO). This clearly indicates that one or several of Mediator’s 21 subunits is SUMOylated. Covalent attachment of SUMO to Mediator thereby preserves this protein from being exchanged during CoIP. SUMOylation of Mediator has been described in a large-scale proteomic study for the identification of SUMOylated yeast proteins^59^.

### The CDK8 Kinase Module Blocks Transcriptional Activity

**Figure 3:**
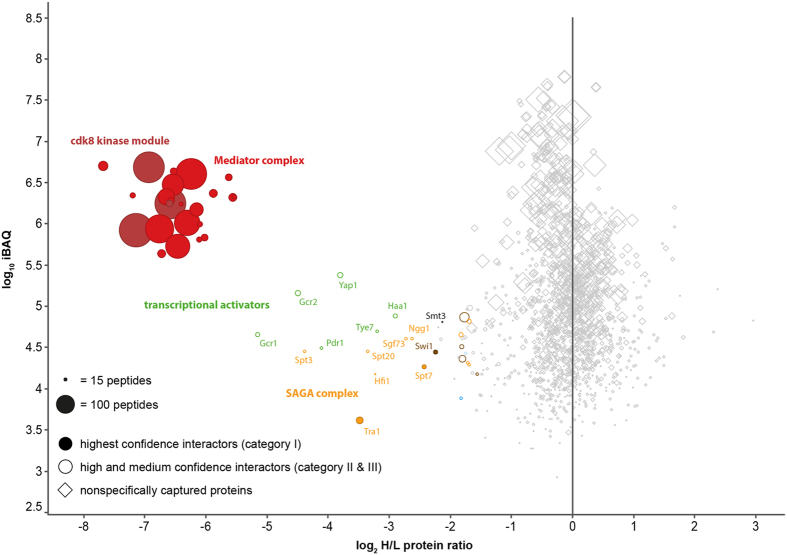
Interactome of the yeast Mediator complex with bound cdk8 kinase module. CoIPs with Med13-HA were performed from cells grown in light medium (^14^N, light), control CoIPs with wild type cells were performed in parallel (mixing after purification, MAP) from metabolically labeled cells (^15^N, heavy). The plotted log_2_-transformed heavy-to-light (H/L) protein ratios and log_10_ iBAQ values are median values calculated from three biological replicates. The complete data set is given in Supplemental Table S1.

The interactome identified from CoIPs with Med17-HA comprises the interaction partners of the 21-subunit Mediator as well as the interactors of the 25-subunit Mediator with bound CKM. In order to figure out which proteins bind to Mediator in the presence of the CKM, and which proteins in the absence of the CKM, we performed experiments with HA-tagged CKM subunit Med13 under the same conditions as for Med17-HA in the MAP experiment. Figure 3 shows that only a very limited number of proteins interact with Mediator in the presence of the CKM, e.g. only six out of the 18 activators. Apart from these activators, a number of subunits of the SAGA complex, as well as a few subunits of the SWI/SNF chromatin remodeling complex are still detected as interaction partners in the presence of the CKM (Fig. 3, Table S3). However, the CKM-bound form of Mediator does neither interact with any of the three RNA polymerases, nor with any of the GTFs or with any of the large number of transcription-related proteins identified from the Med17-HA experiments. Thus, our results demonstrate that essentially all interaction partners associated with active transcription and co-transcriptional processes do not interact with Mediator in the presence of the CKM. These results are well in line with data showing that CKM-containing Mediator represses transcription in yeast^4^. It also becomes particularly evident from this interactome data, that CKM-bound Mediator does not interact with Pol II, as has been derived from structural analyses before^30^. An interaction of Med13 with TFIIS, that has been described recently^60^, is not evident from our data.

## Discussion

Here we present the most comprehensive interactome analysis of the yeast Mediator complex to date. This study greatly enhances results from large-scale interactome analyses in *S. cerevisiae*^21^,^22^ and human cell lines^23^,^24^, that identified only rather stable and high abundant interactors of Mediator. The conditions applied in our study allowed us to identify an extraordinary large number of specific interactors, providing a much more comprehensive picture of Mediator’s cellular environment. One obvious parameter of crucial importance influencing the outcome of an interactome analysis are CoIP buffer conditions^61^. However, other factors such as cell lysis conditions, affinity tag position, and affinity bead type, also significantly influence the number of identified interactors. The conditions finally used for our interactome analyses were the mildest conditions that allowed to preserve the highest number of interaction partners. Different conditions essentially resulted only in a reduced number of identified interactors, without significant complementarity (Figure S5 and data not shown).

Since the interactome of the Mediator complex is highly dynamic and essentially all interaction partners of Mediator are transient, careful optimization of all parameters was exceedingly important for increasing the interactome coverage of our analysis. Since mild CoIP conditions inevitably lead to an increase of protein complexity in CoIP eluates, protein fractionation via SDS-PAGE turned out to be advantageous, and allowed us to reliably distinguish about 450 specific interaction partners from roughly 1200 nonspecifically captured proteins on basis of their H/L protein ratios.

The optimized CoIP conditions applied in our study allowed us to identify many interactions that have escaped detection before. E.g., TFIIB and TFIIF are among the most abundant interactors of Mediator in our data set, whereas the Hahn lab did not detect TFIIF by Western blotting in CoIPs of Med18, and only trace amounts of Mediator in CoIPs with TFIIF^32^. We noticed that co-immunoprecipitating Med17-HA with higher stringency (i.e. 0.5% NP-40 instead of 0.01% NP-40) leads to a significantly decreased number of identified interactors (Figure S5). In particular, TFIIB and one subunit of TFIIF (T2fB) did no longer show up as specific interactors, and the amount of co-purified T2fa, the second subunit of TFIIF, is strongly reduced under these buffer conditions. Furthermore, we recognized that CoIPs with Med18-HA generally result in a much lower number of identified interactors compared to CoIPs with Med17-HA (data not shown), presumably because the affinity tag on Med18 is less well accessible for the antibody compared to Med17. Thus, we think that the discrepancy between the study of the Hahn lab and our study is mainly due to different buffer conditions and to different affinity tag positions.

We have been able to detect a large part of the interaction partners that have been reported for the Mediator complex so far, including even labile interactors, for which the detection was challenging in the past, such as the SWI/SNF chromatin remodeling complex. Moreover, our interactome analysis greatly expands the number of known interactors and reveals potential new, yet uncovered functions of the Mediator complex. Besides essentially all proteins of the PIC, reflecting Mediator’s major function of activator-dependent promotion of PIC assembly, we identified for example a number of proteins involved in elongation and in co-transcriptional processes, such as splicing, mRNA decapping and mRNA decay, indicating that Mediator is involved in these co-transcriptional processes, too. Probably the most unexpected and exciting result of our interactome analysis is that Mediator interacts not only with Pol II, but also with Pol I and Pol III, as well as with a number of proteins involved in rRNA processing and ribosome biogenesis. This indicates that Mediator’s functional role might not be limited to mRNA transcription, but extends to any type of RNA transcription.

The methods and conditions applied in this study allowed for an extraordinary deep interactome analysis, and many new interactors of Mediator have been identified, from which we have discussed only the most prominent ones. Many more interactors, such as DNA topoisomerase 2, which has been shown to be involved in transcription regulation^62^, and other transcription-related proteins, kinases such as Ksp1, proteins involved in telomere length regulation (e.g. Tel2), and many others more have been identified in this work (Table S1). Thus, we think that the comprehensive list of specific interaction partners obtained from this study is a valuable resource for generating new hypothesis about the Mediator complex and its diverse cellular functions.

## Methods

### Chemicals and Proteases

(^15^NH_4_)_2_SO_4_ (>99% isotopic purity) was obtained from Cambridge Isotope Labs, trypsin (gold, MS grade) was ordered from Promega. All other reagents were obtained from Sigma-Aldrich.

### Yeast Strains

Wild type strain S288c (MATα SUC2 gal2 mal2 mel flo1 flo8-1 hap1 ho bio1 bio6) (LGC Standards) was used to generate HA-tagged strains S288C_Med17-6HA (MATα SUC2 gal2 mal2 mel flo1 flo8-1 hap1 ho bio1 bio6 HygMX Med17-6HA) and S288C_Srb9-6HA (MATα SUC2 gal2 mal2 mel flo1 flo8-1 hap1 ho bio1 bio6 HygMX Srb9-6HA).

### PCR-based C-terminal Epitope Tagging

DNA fragments were amplified using Pwo Polymerase PCR reaction mix (PEQ Lab) and the vector pYM16 as template as recently described^63^. PCR conditions were as follows: a 95 °C for 5 min denaturation step was followed by 30 cycles of 30 s at 95 °C, 30 s at 54 °C, and 2 min at 68 °C. PCR products were purified and 3–4 μg used for transformation in competent *S. cerevisiae* cells^64^. Transformed cells were plated on agar plates containing hygromycin (300 μg/mL) as selection marker. Integration of the PCR-fragment by homologous recombination was confirmed by PCR and sequencing. Proper expression and ability of purification of the tagged Mediator subunit was validated by immunoprecipitation and Western blotting.

### Metabolic Labeling, Cell Lysis and Co-Immunoprecipitation

For stable isotope labeling yeast cells were grown in yeast minimal medium (1.7 g yeast nitrogen base without amino acids and ammonium sulfate (VWR), 20 g glucose) containing 1 mg/L (^14^NH_4_)_2_SO_4_ as exclusive nitrogen source for HA-tagged strains and 1 mg/L (^15^NH_4_)_2_SO_4_ for the wild type strain. Standard growth conditions were applied (30 °C, 230 rpm) and cells were harvested at OD_600_ = 0.7–0.8. Cryogenic disruption in a PM 100 planetary ball mill (Retsch) was performed according to the protocol from the Rout lab^25^, and 1 g cell powder was thawed in 5 mL lysis buffer (20 mM HEPES pH 7.5, 300 mM KOAc, 10% Glycerol, 0.01% 4-Nonylphenyl poly(ethylene glycol) (NP-40), 6 mM MnSO_4_, 1 mM DTT, 50 U/mL Cyanase (SERVA)) and mixed with four strokes in a Dounce tissue grinder. Cell debris was centrifuged at 20,000 g (Avanti J-26XP, Beckmann Coulter) at 4 °C for 20 min, and the supernatant was used for CoIP.

CoIPs were performed with Pierce anti-HA magnetic beads (Thermo Scientific) for 2 h at 4 °C and slow rotation. Magnetic beads were washed four times with lysis buffer, and eluted with LDS sample buffer (Invitrogen) without reducing agent (15 min, 37 °C at 400 rpm). Eluates resulting from the CoIPs with and without epitope tag were pooled and acetone precipitated (5-fold volume of acetone) together overnight at −20 °C.

### SDS-PAGE and Tryptic Digest

Precipitated proteins were dissolved in 25 μl LDS sample buffer (Invitrogen) containing 50 mM DTT and heated for 10 min at 70 °C. Cysteine residues were alkylated with 120 mM iodoacetamide for 20 min at room temperature, and proteins were separated on NuPAGE Novex 4–12% Bis-Tris gels (Life Technologies) with MOPS buffer according to manufacturer’s instructions. Gel staining was performed with SimplyBlue (Life Technologies) according to manufacturer’s instructions. Gel lanes were cut into 15 bands, and gel bands were chopped and destained with 70% acetonitrile in 100 mM NH_4_HCO_3_ (pH 8), shrunk with 100% acetonitrile and dried in a vacuum concentrator (Concentrator 5301, Eppendorf). Dried gel pieces were suspended in 100 mM NH_4_HCO_3_ (pH 8) containing 0.1 μg trypsin. Digests were performed overnight at 37 °C, and peptides were extracted from the gel slices with 5% formic acid and transferred to LC vials.

### NanoLC-MS/MS

NanoLC-MS/MS analyses were performed on an Orbitrap Fusion (Thermo Scientific) equipped with an EASY-Spray ion source and coupled to an EASY-nLC 1000 (Thermo Scientific). Peptides were loaded on a trapping column (2 cm × 75 μm ID. PepMap C-18 3 μm particles, 100 Å pore size) and separated on an EASY-Spray™ column (25 cm × 75 μm ID, PepMap C-18 2 μm particles, 100 Å pore size) with a 30-minute linear gradient from 2% to 32% acetonitrile and 0.1% formic acid. MS and MS/MS scans were acquired in the Orbitrap analyzer with a resolution of 60.000 and 15.000, respectively. HCD fragmentation with 35% normalized collision energy was applied. A Top Speed data-dependent MS/MS method with a fixed cycle time of 3 seconds was used, and dynamic exclusion was applied with an exclusion duration of 30 seconds. Minimum signal threshold for precursor selection was set to 50,000. Predictive AGC was used with AGC a target value of 2 × 10^5^ for MS scans and 5 × 10^4^ MS/MS scans. EASY-IC was used for internal calibration. All raw data files are publicly available via Chorus under ID 1142 (https://chorusproject.org/).

### Processing and Database Search

For raw data processing, database searches and quantification, Mascot Distiller 2.5.1 in conjunction with Mascot Server 2.5.1 (Matrix Science) were used. Raw data files (i.e. 45 LC-MS/MS runs per triplicate) were processed as a multi-file project with Mascot Distiller essentially with the supplied standard settings for Orbitrap data (high/high). Mascot database searches were performed against the *S. cerevisiae* S288c UniProt reference proteome (ID: UP000002311, 6743 entries, download date: 20150502, reverse concatenated) and a contaminant database (245 entries). The following search parameters were used: Trypsin cleavage with up to 3 missed cleavages; fixed modification: Carbamidomethyl (C); variable modifications: Acetyl (Protein N-term), Gln- >pyro-Glu (N-term Q), Oxidation (M); peptide mass tolerance: 10 ppm and fragment mass tolerance: 0.02 Da. Mascot Distiller setting for quantification included a ^15^N incorporation set to 99.5% for heavy labeled peptides, a minimum peptide score for quantitation of 15 and inclusion of charge-matched peptides for quantification.

PEAKS 7.5 (Bioinformatics Solutions) was used for the analysis of histone acetylation with the following parameters: parent mass tolerance: 8 ppm; fragment mass tolerance: 0.02 Da; enzyme: trypsin; missed cleavages: 5; non-specific cleavage: none; fixed modifications: Carbamidomethyl (C); variable modifications: Acetyl (Protein N-term), Gln- >pyro-Glu (N-term Q), Oxidation (M), Methyl (K & N-terminal peptide); Dimethyl (K & N-terminal peptide), Trimethyl (K & N-terminal peptide); Acetyl (K & N-terminal peptide); phosphorylation (STY); ubiquitination (K); max. variable modifications per peptide: 8; database: *S. cerevisiae* S288c UniProt reference proteome (ID: UP000002311, 6743 entries, download date: 20150502, reverse concatenated). PEAKS results were filtered with 0.5% peptide FDR.

### Mascot Results FDR filtering

The complete quantification results were exported from Mascot Distiller as XML file. For all further analysis steps, in house developed R scripts were used. Peptide ratios and protein identifications were extracted from the XML file. Peptides were FDR filtered (1%) utilizing reverse hits from the target-decoy database, and only peptides with a Mascot search rank of 1 were kept. For protein identification, at least 2 unique peptides were required, and proteins quantified in less than two replicates were excluded.

### Calculation of Quantitative Values and Statistical Analysis

Three biological replicates were performed for all CoIP experiments. The wild type strain, grown in ^15^N-medium (heavy) was used in all experiments for control CoIP. Mixing was performed either before (PAM experiment) or after CoIP (MAP experiment).

Separately for each replicate experiment, the median of all log_2_-transformed H/L peptide ratios for each protein and the log_10_-transformed sum of heavy and light peptide intensities as well as iBAQ values^28^ were calculated. Resulting protein ratios were centered to the first mode of the distribution. Significantly enriched proteins were identified by using robust statistics based on intensity binned boxplots (at least 300 proteins per bin), proteins outside the 1.5- or 3-fold interquartile range (IQR) of the box were classified as significant (1) or extreme significant (2) outliers, respectively. Proteins with high variation in their peptide ratios were considered as not significant. For this purpose, a boxplot was calculated for each protein of the corresponding intensity bin and it was checked, that the individual boxplot notch (1.58-fold IQR divided by square root of the number of quantified peptides) was not overlapping with the median ratio (around zero) plus or minus the median of all notches.

For combining the replicates, the protein ratios were quantile normalized^65^ and median ratios were calculated from the three replicates for each protein. These were centered, and boxplot statistics was calculated (as described above).

Proteins identified as extreme significant outliers (2) in the combined replicate data (triplicates), were further categorized as follows: in category I (highest confidence interactors) are proteins that have been identified as extreme significant outlier in at least two replicates and as significant outlier in the third replicate (2-2-2 or 2-2-1). Proteins of category II (high confident interactors) have been identified as extreme significant outliers in at least one replicate and at least as significant outlier in at least another replicate (2-2-0, 2-1-1, or 2-1-0) or as significant outlier in all three replicates (1-1-1). In category III (medium confident interactors) are all other proteins that have been identified as extreme significant outlier in the triplicate data.

Comprehensive data tables containing all identified and quantified peptides and proteins together with all necessary information are given in Supplemental Material for all experiments.

## Additional Information

**How to cite this article:** Uthe, H. *et al*. Proteomic Analysis of the Mediator Complex Interactome in *Saccharomyces cerevisiae. Sci. Rep.* **7**, 43584; doi: 10.1038/srep43584 (2017).

**Publisher's note:** Springer Nature remains neutral with regard to jurisdictional claims in published maps and institutional affiliations.

## Supplementary Material

Supplementary Figures

Supplementary Table 1

Supplementary Table 2

Supplementary Table 3

## Figures and Tables

**Table 1:** Selection of interactors of the Mediator complex (functionally classified).

*Pol II-related*
Pol II (12/12): Rpab1-5, Rpb1-4, 7, 9, 11
**Activators**
Gcr1, Gcr2, Yap1, Gcn4, Pdr1, Hsf1, Skn7, Asg1, Crz1, Fzf1, Met4, Met32, Gat1, Tye7, Msn4, Haa1, Cup2, Bas1
MBF (2/2): Swi6, Mbp1
**Initiation**
TFIIB (1/1): Tf2b
TFIID (15/15): Taf1-14, Tbp
TFIIE (2/2): T2ea, T2eb
TFIIF (3/3): T2fa, T2fb, Taf14
TFIIH (6/10): Tfb1-3, Rad3, Rad25, Ccl1
SAGA (19/20): Taf5, 6, 9, 10, 12, Tra1, Hfi1, Spt3, 7, 8, 20, Gcn5, Ada2, Sgf29, Ngg1, Ubp8, Sgf11, 73, Sus1
Sub1
**Elongation and co-transcriptional processes**
FACT (2/2): Spt16, Pob3
Spt6
Tfs2, Pabp
Rtr1 (CTD phosphatase), Brr2 & Cbp2 (splicing), Dcp2 & Edc3 (mRNA decapping), Xrn1 (mRNA decay)
***Pol I-related***
Pol I (14/14): Rpa1-2, 12, 14, 34, 43, 49, Rpab1-5, Rpac1-2
**Initiation**
UAF (1/6): Rrn5
**Elongation**
RENT (3/3): Net1, Cdc14, Sir2
Hmo1
**rRNA processing & ribosome biogenesis**
Mdn1, Dbp7, 10, Drs1, Enp1, Mak5, Noc3, Nop2, 4, 13, 14, Rpf2, Utp20, 22
***Pol III-related***
Pol III (6/17): Rpc1-2, Rpab1-4
**Initiation**
TFIIIB (2/3): Tf3b, Tbp
TFIIIC (2/6): Tfc1,3
***Chromatin remodeling***
SWI/SNF (10/11): Swi1, 3, Snf2, 5, 6, 12, Arp7, 9, Swp82, Taf14
RSC2 (9/17): Rsc3, 4, 6, 7, 9, Arp7, 9 Sth1, Rt102
INO80 (4/15): Ino80, Ies1, Arp4, Taf14
SWR1 (3/16): Swc4, Bdf1, Arp4
ISW1b (2/3): Isw1, Ioc2
ISW2 (1/2): Isw2
***Histones***
H2A1, H2B2, H3, H4, H2AZ
***Others***
Ty1 polyproteins: Yo11a, Yn12a, Yo11b, Yg11b, Ye12b, Yb11b, Yc21b, Yl21b
Inosine-5′-monophosphate dehydrogenases (IMDHs): Imdh1-4
Importins: Ima1, Imb1,3,4, Sxm1
Pex19
