# Supplementary material for: Proteomic Analysis of the Mediator Complex Interactome in Saccharomyces cerevisiae
Source: Sci Rep. 2017 Feb 27;7:43584. doi: 10.1038/srep43584 (PMC5327418; doi:10.1038/srep43584)
Supplement: Supplementary Figures [file srep43584-s1.pdf]

Supplementary Figures S1-S5

Proteomic Analysis of the Mediator Complex Interactome in *Saccharomyces cerevisiae*

Henriette Uthe, Jens T. Vanselow and Andreas Schlosser*

Rudolf Virchow Center for Experimental Biomedicine, University of Wuerzburg,

Josef-Schneider-Str. 2, 97080 Wuerzburg, Germany

* To whom correspondence should be addressed. Email: andreas.schlosser@uni-wuerzburg.de

A

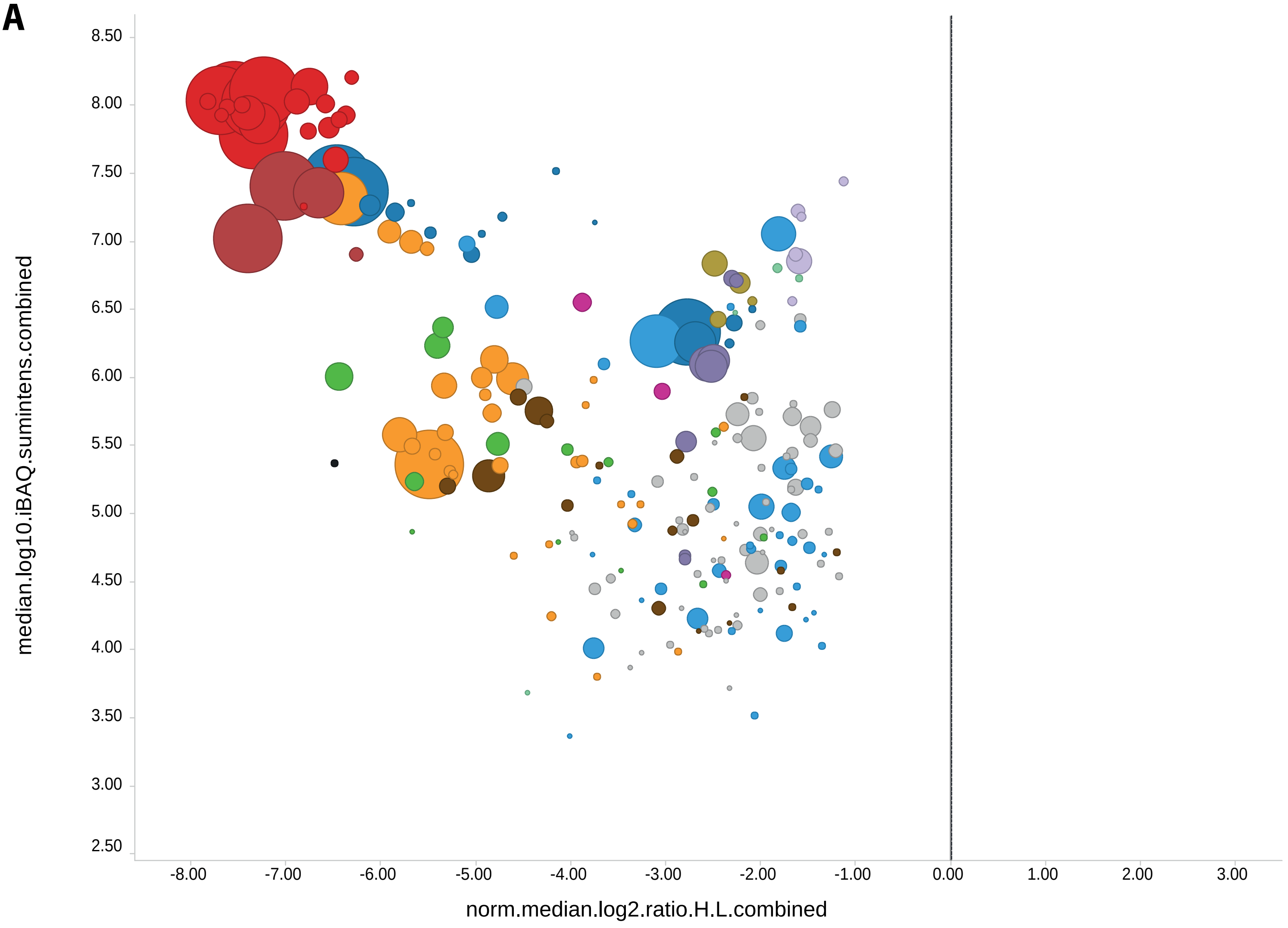

median.log10.iBAQ.sumintens.combined
8.50
8.00
7.50
7.00
6.50
6.00
5.50
5.00
4.50
4.00
3.50
3.00
2.50
-8.00
-7.00
-6.00
-5.00
-4.00
-3.00
-2.00
-1.00
0.00
1.00
2.00
3.00
norm.median.log2.ratio.H.L.combined

B

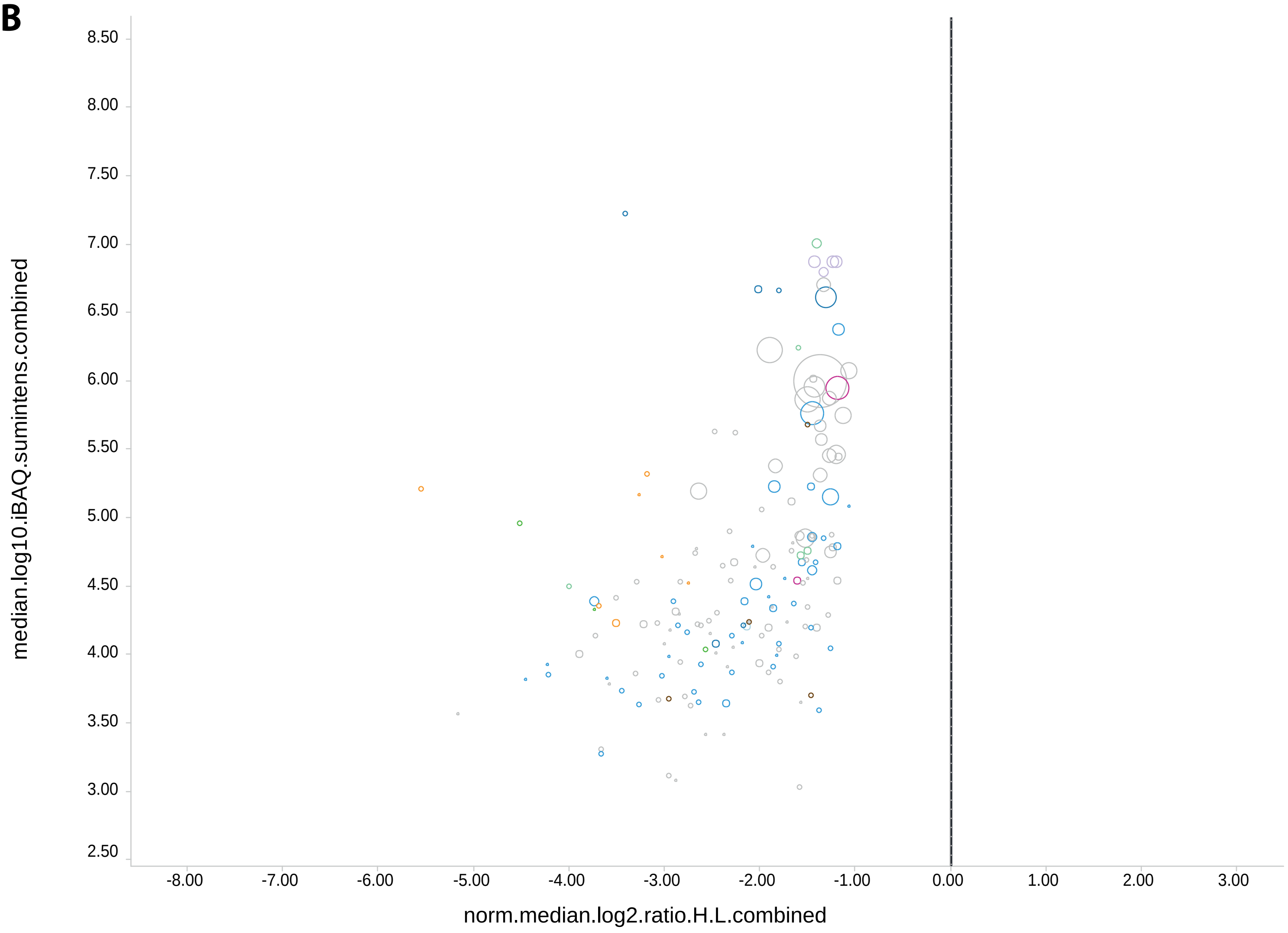

8.50
8.00
7.50
7.00
6.50
6.00
5.50
5.00
4.50
4.00
3.50
3.00
2.50
median.log10.iBAQ.sumintens.combined
-8.00
-7.00
-6.00
-5.00
-4.00
-3.00
-2.00
-1.00
0.00
1.00
2.00
3.00
norm.median.log2.ratio.H.L.combined

C

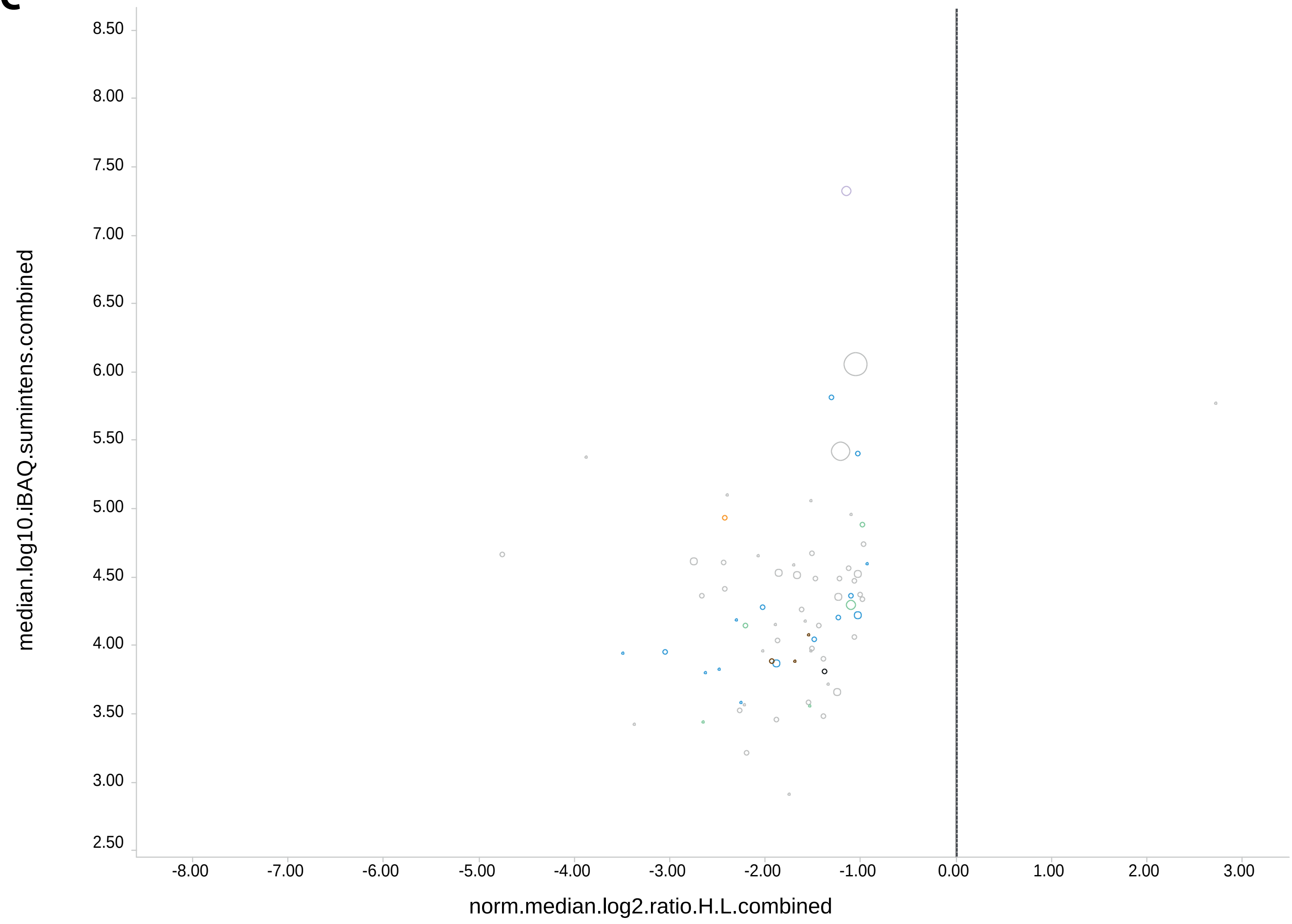

Figure S1: The 467 identified interaction partners of the yeast Mediator complex as shown in Figure 1, but separated by the three different categories. **A.** 228 interactors of category I. **B.** 167 interactors of category II. **C.** 72 interactors of category III (see also Table S1 for details).

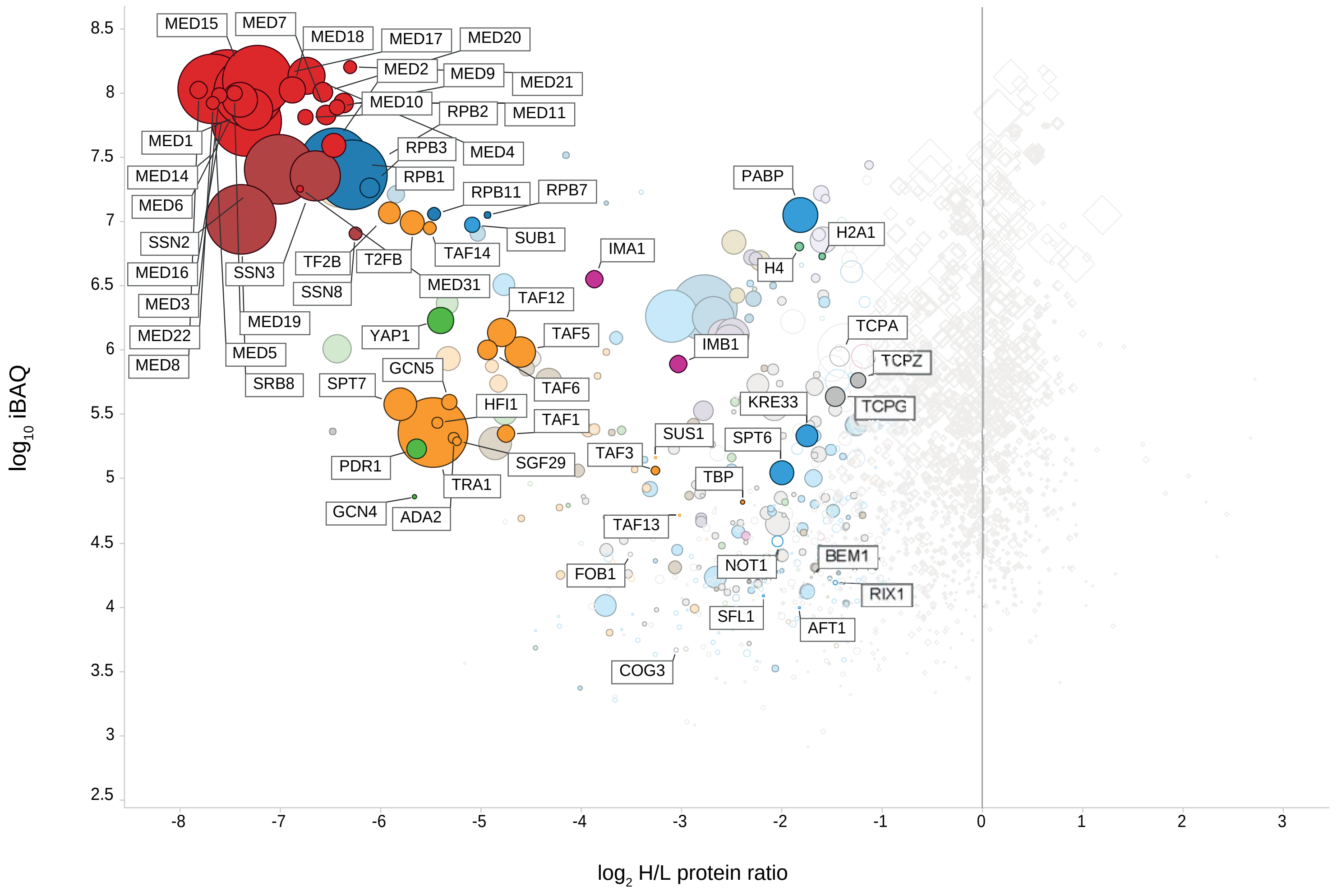

Figure S2: Interactome data from Figure 1 with all proteins marked that are listed as high confident interaction partner in the HitPredict database (see also Table S1).

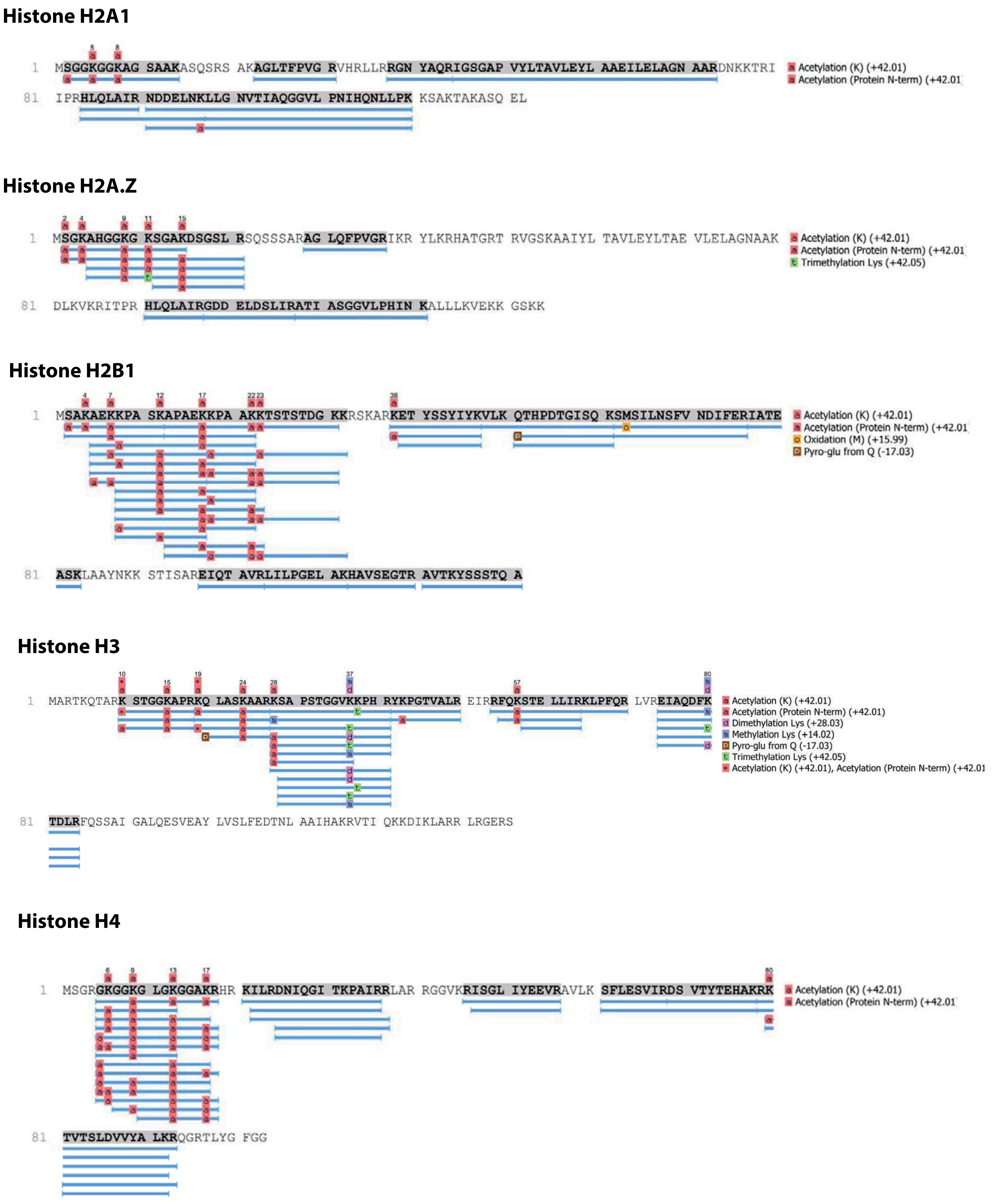

Figure S3: Sequence coverage of histones H2A, H2A.Z, H2B, H3 and H4, which have been identified as specific interactors of the Mediator complex (see Figure 1). All histones are highly acetylated at their N-termini. Database search was performed with PEAKS 7.5, and peptides were filtered with 1 % FDR.

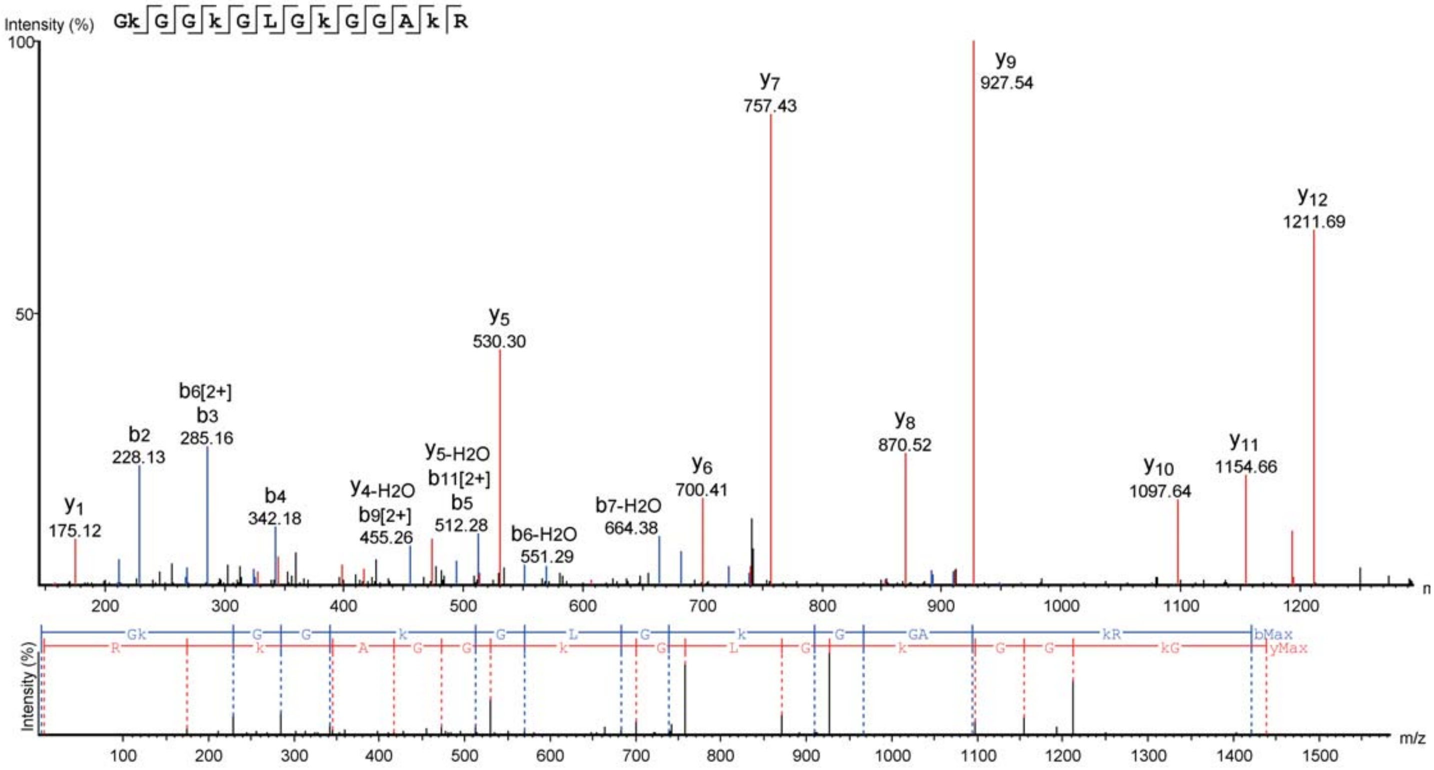

Figure S4: Annotated fragment ion spectrum (HCD spectrum) of the tryptic peptide GKGGKGLGKGGAKR of the histone H4 indicating acetylation of all four lysine residues.

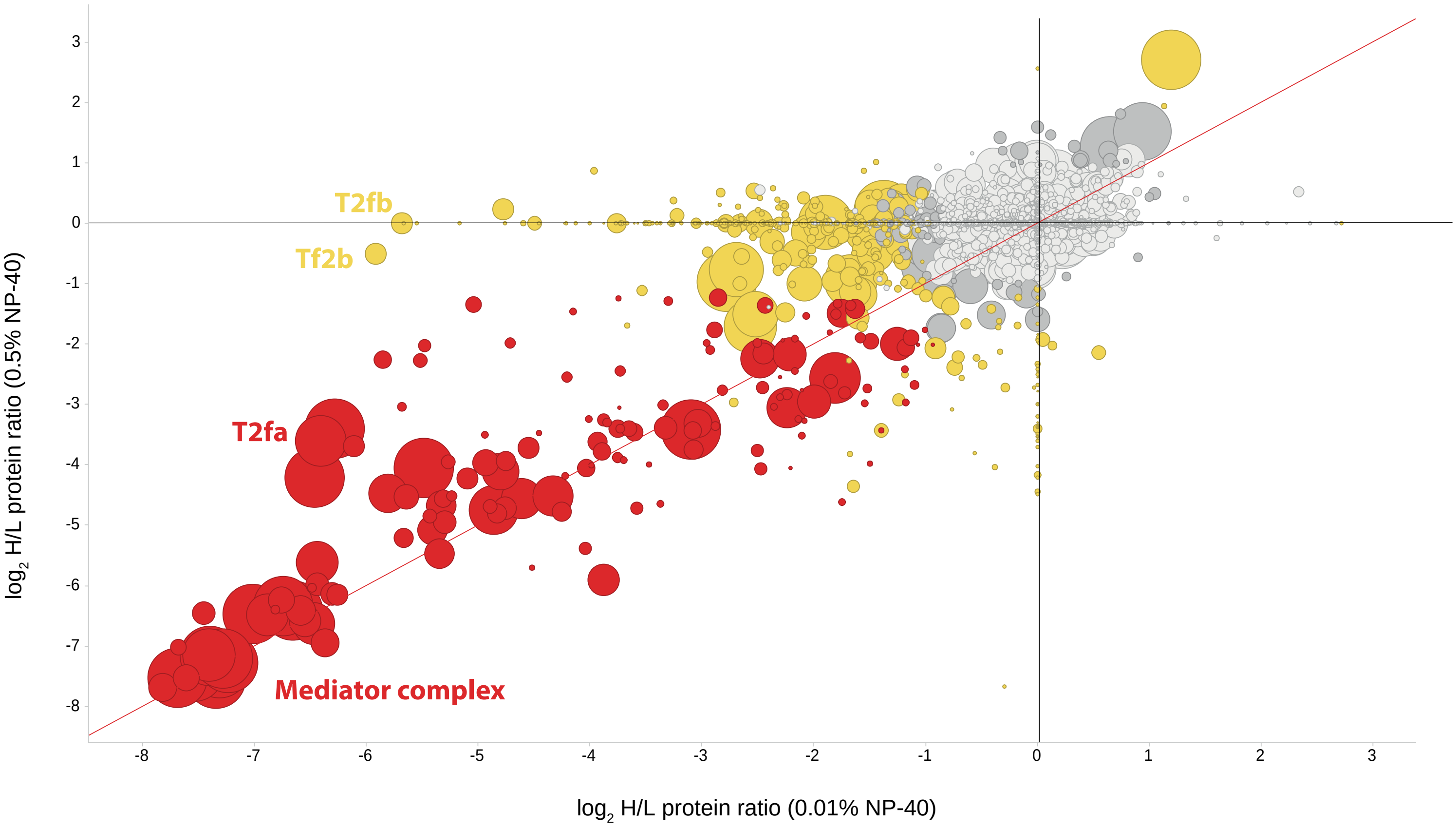

Figure S5: Triplicate Med17-HA CoIP experiments performed with 0.01% NP-40 and with 0.5% NP-40. The $\log_2$ H/L protein ratios from the two experiments are plotted against each other. Significantly less interactors are identified with 0.5% NP-40. Colors: red: identified as specific interactor in both experiments; yellow: identified as specific interactor in only one experiment; grey: unspecific binder in both experiments.
